# Using life and death education to guide the teaching and research of mindfulness

**DOI:** 10.3389/fpubh.2025.1576500

**Published:** 2025-06-16

**Authors:** Huy P. Phan, Bing Hiong Ngu, Si-Chi Chen, Chao-Sheng Hsu

**Affiliations:** ^1^School of Education, University of New England, Armidale, NSW, Australia; ^2^Department of Education, National Taipei University of Education, Taipei, Taiwan

**Keywords:** mindfulness, meditation, life education, death education, humanistic life experiences, altruistic life ideals

## Abstract

Our teaching and research undertakings, facilitated by extensive international collaboration and networking, have led us to recently proposed an alternative conceptualization of mindfulness. For example, mindfulness has been predominantly interpreted through a psychological lens, often defined as an individual's capacity to be non-judgmental in the present moment. This perspective, we acknowledge, is limited as it fails to acknowledge and/or take into account the broader philosophical and spiritual dimensions of mindfulness. Our reconceptualization largely coincides with the principles of *life* and *death education* teaching, which delve into the humanistic nature of life. Life and death education encourages introspection into humanistic pursuits, such as the aspiration to achieve altruistic life ideals (e.g., a teenager's wish to help others). Such personal practices, we contend, may resonate with the deeper philosophical and spiritual teachings of mindfulness, which emphasize the importance of kindness, generosity, compassion, and similar values. Our conceptualization, as discussed in the present theoretical-conceptual article, proposes a reciprocal relationship between mindfulness and life and death education. We argue that life and death education may provide theoretical grounding to complement the teaching of mindfulness and, likewise, the nature of mindfulness may facilitate a deeper engagement with the principles taught in life and death education. Furthermore, in its current stage of development, our theoretical-conceptual premise remains formative, grounded primarily in philosophical analysis and preliminary integrative teaching practices. While conceptually promising, we recognize the present limitations in empirical validation. Accordingly, this article is intended to serve as a foundational platform to stimulate scholarly discourse and guide future lines of inquiry, including empirical examination and curriculum development. We posit that the integration of mindfulness with life and death education holds considerable potential—not only for advancing academic scholarship but also for fostering individual wellbeing, spiritual insight, and the cultivation of humanistic life ideals.

## 1 Introduction

*Mindfulness* is an interesting area of study and practice in the social sciences (1–3), enriched by its diverse interpretations and applications. Its multifaceted nature (4–6) stems from the various philosophical and cultural frameworks through which it is understood. Our teaching and research primarily draw from Eastern philosophies, such as Buddhism (7, 8), Confucianism (9, 10), and Taoism (11), offering insights into mindfulness as both a mental discipline and a way of life. This interdisciplinary approach highlights the depth and adaptability of mindfulness, making it a rich subject for academic exploration and practical application (e.g., the use of “walking meditation” to calm one's state of attention). Building on the principles of mindfulness, our research work extends to the closely related field of “life and death education” (12–14). Through in-depth teaching and research, we explore this subject, which similarly emphasizes *awareness, introspection*, and the *cultivation* of meaningful perspectives on existence and mortality.

An interesting proposition we explore in this article is whether, and to what extent, *life* and *death education* (12–14) teaching can complement the study of mindfulness. For example, we posit that life and death education may deepen mindfulness by addressing existential themes (e.g., the nature of spirituality), fostering a more profound awareness of life's impermanence (e.g., the endless cycle of birth-death-rebirth), and promoting emotional resilience and spiritual growth. Over the past two decades, our teaching practices have embraced what we term “theoretical infusion” (15)—integrating mindfulness teaching with the thematic content of life and death education (e.g., emphasizing the nature of “proactive life functioning”) and reciprocally incorporating themes from mindfulness (e.g., a focus on the perceived feeling of “self-enlightenment”). We argue that this pedagogical approach is effective and enriches the theoretical understanding of both disciplines. Consequently, our inquiry considers an engaging premise: that life and death education teaching may advance the nuanced complexities and foundational nature of mindfulness.

The present theoretical-conceptual article seeks to advance research into the intricate relationship between mindfulness and life and death education. We propose that these two theoretical frameworks share a deeply interconnected, reciprocal relationship. Life and death education teaching (12–14) offers valuable insights into the fundamental nature of mindfulness (1, 5, 16), situating its principles within the broader context of human existence. That in-depth understanding of proactive life functioning, for example, may help a person appreciate the practice of meditation and/or the perceived sense of mindfulness. Similarly, an understanding of mindfulness (e.g., the meaning of being non-judgmental) may facilitate a deeper comprehension of life's true meaning. We hope that our analysis and subsequent proposition will contribute to the advancement of both teaching and research in mindfulness, with a focus on utilizing life and death education as a theoretical foundation.

## 2 Mindfulness: an introduction

*Mindfulness* is a theoretical concept with numerous definitions, reflecting a wide range of viewpoints, interpretations, and perspectives [e.g., (1, 5, 17, 18)]. As a research team, international in makeup, we differ in our teaching approaches, understandings, and interpretations of mindfulness—a topic we examine in greater depth in the following discussion. This “diversity”, we contend, may help contribute to the understanding of the multifaceted nature of mindfulness itself. For instance, one co-author approaches mindfulness through a psychological lens, emphasizing non-cognitive and emotional processes (e.g., regulating a state of calmness). In contrast, two co-authors emphasize its spiritual dimensions, highlighting connections between mindfulness, the sacred, and the transcendent.

Mindfulness, as commonly defined in Western literature, is described as “moment-to-moment, non-judgmental awareness, cultivated by paying attention in a specific way—that is, in the present moment, and as non-reactively, non-judgmentally, and openheartedly as possible” (1). Meiklejohn, Phillips (19) similarly define mindfulness as a state of consciousness and heightened self-awareness that deepens over time. It is a psychological construct that highlights the significance of the “personal self”, encompassing qualities such as contemplation, internal self-awareness, concentration, and relaxation. This mechanism underscores the importance of personal, emotional, and physiological functioning. Notably, mindfulness serves as a counterbalance to life-related negativities, including angst, pessimism, and mindlessness (16).

At first glance, the above sample definitions appear broadly accepted and credible. One of the co-authors, with a specialization in Educational Psychology, affirms that Kabat-Zinn's (1) conceptualization of mindfulness is both valid and relevant within contemporary educational and psychological contexts. In a similar vein, extensive research has employed surveys and Likert-scale measures to assess and evaluate the effectiveness and nature of mindfulness as conceptualized within the framework of Western interpretations. These studies often focus on quantifying mindfulness-related outcomes, such as academic achievement, emotional regulation, stress reduction, and overall wellbeing, aligning with definitions like Kabat-Zinn's. Such methodological approaches (e.g., a quantitative methodological approach using structural equation modeling) provide valuable data for validating the potency of meditation practices and their applications across diverse contexts (20).

A significant limitation of the Western framework for understanding mindfulness is its predominant focus on its psychological aspects. This framework often highlights non-judgmental awareness of the present moment as a defining characteristic of mindfulness, emphasizing the individual's subjective experience. Additional traits, such as cultivating calmness and reducing negative emotions like anxiety and fear, are similarly framed within the context of emotional self-regulation. While this approach provides valuable insights into the psychological benefits of mindfulness, it may overlook broader dimensions, such as *cultural, philosophical*, and *spiritual* contexts, that could enrich its conceptualization and application. As an introduction to a personal narrative supporting this claim, one of the co-authors presented his research and perspective on mindfulness at a workshop in Taiwan with Taiwanese scholars in 2016. His presentation received polite critique from several scholars, who expressed that the co-author's interpretation leaned heavily toward a psychological emphasis, which they considered somewhat biased. Additionally, some Taiwanese scholars specializing in Buddhism highlighted an important distinction between “mindfulness” and “meditation”. They explained that successful meditation practices, such as “walking meditation”, enable individuals to achieve a state of mindfulness. In other words, meditation is *a practice*, while mindfulness is the *resulting state* characterized by specific attributes. They argued that conflating the two terms is not only improper but also misleading.

### 2.1 Existing research development

An analysis of the existing literature reveals a plethora of research, both theoretical and empirical, examining the potency and applicability of mindfulness [e.g., (21–25)]. Central to this body of work is a growing collection of evidence demonstrating the positive effects of mindfulness. Specifically, engagement in mindfulness has been shown to promote positive emotions, foster a stable temperament, and enhance overall wellbeing. Specifically, successful mindfulness practice may produce:

- A clear and balanced state of mind, associated with personal contentment and physiological calmness, ease, and clarity.
- Enhanced emotional functioning, such as increased happiness and reduced anxiety, contributing to improved academic and non-academic performance.

Educationally, mindfulness has been shown to positively impact learning and achievement outcomes across diverse contexts (26–28). These findings have informed the development of various educational-social programs aimed at fostering mindfulness skills, such as the Master Mind Program (29). In their review, Meiklejohn et al. (19) identified programs worldwide designed to teach and encourage mindfulness practice in education, benefiting both teachers and students. For example, the Mindfulness Education (ME) Program (30) focuses on fostering emotional and social competence through mindfulness lessons, promoting optimism and positive affect. Adolescents participating in the program demonstrated improved optimism, supporting the value of such interventions. Key benefits include:

i. For teachers:
  - Cultivation of mindfulness in daily life, enhancing wellbeing.
  - Improved emotional and instructional support for students.
  - Strengthened engagement and professional motivation.
  - Reduction in anxiety and negative emotions.

ii. For students:
  - Improved self-regulation of attention.
  - Better emotional regulation and reduction in behavioral problems.
  - Increased social skills, academic performance, and optimism.
  - Enhanced feelings of calmness, relaxation, and self-acceptance.

The findings from the above underscore the importance of ongoing research into the effectiveness and efficacy of mindfulness-based interventions. Meiklejohn et al. (19) recommend further exploration to validate interventions, understand their underlying mechanisms, and identify the conditions in which they are most effective. As an example, practicing meditation prior to stress-inducing situations, such as formal exams, may be beneficial for students. The authors propose advancing mindfulness research through the development of robust theories of change, expanding evidence bases, validating outcome measures, and addressing implementation challenges in schools. Such efforts are crucial for establishing mindfulness as a distinct theoretical framework, supported by robust evidence, and ensuring its broader impact across various domains of practice. For instance, our recent research aimed to link different aspects of mindfulness—such as personal experiences of self-enlightenment (31–33)—with the *paradigm of positive psychology* (34–36).

## 3 A multifaceted approach of mindfulness: our proposition

Since one of the co-authors' workshop presentations in 2016, the research team has been involved in an international research collaboration focusing on various aspects of human agency, including *mindfulness* and *meditation* [e.g., (37)], *optimal functioning* [e.g., (38)], and *life* and *death education* [e.g., (15)]. Several years ago, the research team published an article critically analyzing the Western perspective on mindfulness. We argued that such interpretations—such as Kabat-Zinn's (1) definition and viewpoint—are limited and fail to capture the full scope and complexity of mindfulness. Our proposal, in contrast, views mindfulness as a multifaceted construct composed of key latent factors and corresponding attributes. It is important to note, however, that our use of the terms “multifaceted” and “multidimensional” differs slightly from their traditional, quantitative interpretation. Several researchers have developed inventories and scales to measure and assess the multidimensional structure of mindfulness. These include: the Five *Facet Mindfulness Questionnaire* (FFMQ) (4, 5), the *Mindful Attention and Awareness Scale* (MAAS) (39, 40), the *Toronto Mindfulness Scale* (TMS) (41, 42), *The Revised 12-item Cognitive and Affective Mindfulness Scale* (CAMS-R) (43), *The Southampton Mindfulness Questionnaire* (SMQ) (44, 45), *T*he Philadelphia Mindfulness Scale (PHLMS) (6, 46), the 30-item *Freiburg Mindfulness Inventory* (FMI) (47, 48), the *Kentucky Inventory of Mindfulness Skills* (KIMS) (18, 49), the *Mindfulness-Based Relapse Prevention Adherence and Competence Scale* (MBRP-AC) (50, 51), the *Self-Comparison Scale* (S-CS) (52, 53), and the *Self-Other Four Immeasurables* (SOFI) (54, 55). Some inventories, such as the FFMQ (4, 5), consider the importance of a multifaceted representation of mindfulness, while others adopt a unitary theoretical approach.

Our proposition differs significantly by adopting a broader perspective, emphasizing the *philosophical, psychological*, and *spiritual* dimensions of mindfulness (20, 37). Existing research inquiries from Western contexts have primarily emphasized the psychological aspects of mindfulness, focusing on constructs such as an individual's perception of judgment and emotional self-regulation [e.g., (5, 6, 56)]. However, this interpretation is somewhat narrow and incomplete, as it overlooks the foundational teachings of Eastern philosophy, particularly Buddhism (7, 8, 57), where mindfulness originates. For instance, the Pali term *sati*, often translated as “mindfulness”, conveys a more holistic understanding that is integral to the practice and philosophy of Buddhism.

Our proposition of the multifaceted structure of mindfulness, as outlined, encompasses three distinct dimensions: philosophical, psychological, and spiritual (20, 58). Each dimension, such as the philosophical dimension, is characterized by specific defining attributes—for example, the philosophical dimension highlights the principle of *rationality*. We propose that integrating these three dimensions into a unified theoretical model of mindfulness offers a comprehensive framework for understanding the full spectrum of human experiences. Our multifaceted approach not only embraces the complexity of mindfulness but also integrates diverse theoretical perspectives and interpretations, recognizing the interconnectedness of psychological processes, philosophical insights, and spiritual beliefs. In this analysis, we argue that traditional assessment tools, such as Likert-scale measures and similar methodological approaches (59–61), fall short in capturing the nuanced and complex nature of mindfulness. These instruments and diagnostic tools, in fact, may reduce rich, multidimensional phenomena to simplified, quantified metrics that overlook the intricacy and “totality” of mindfulness.

Building on our previous discussion, we emphasize the crucial relationship between mindfulness and meditation practice. As previously mentioned, we draw upon our interpretation and perspective of Eastern philosophy to suggest that engaging in meditation—such as traditional Buddhist meditation practices (57, 62)—can cultivate and sustain a state of mindfulness. This connection underscores how meditation serves as a foundational mechanism for developing mindfulness, facilitating heightened awareness, focused attention, and a deepened sense of presence. We succinctly denote this relationship as follows: *practice* of meditation → *state* of mindfulness, where “ → ”indicates a potential causal link. In this regard, meditation practice is a deliberate and beneficial activity that plays a key role in fostering a state of mindfulness. Furthermore, to emphasize our conceptualization of a multifaceted structure, we argue that a state of mindfulness encompasses a wide range of human experiences, including:

**i. Psychological processes of information processing**

This reference pertains to the *psychological mechanisms* that govern how an individual perceives, interprets, stores, and retrieves information. In the context of mindfulness, however, these psychological processes extend beyond simple cognition to encompass an understanding of one's state of awareness, thoughts, emotions, and more in the present moment. What is the person sensing in this very moment? Are they experiencing calmness, or is it some convoluted information that is difficult to process? We contend that meditation practice, such as Buddhist meditation (57, 62), enables a person to experience a perceived sense of mindfulness, enhancing their ability to stay focused and attentive to different contextual influences. Furthermore, as existing viewpoints suggest, engaging in such practices can lead to notable improvements in clarity of perception and emotional regulation. For example, a 15-min “walking meditation” practice may help instill a sense of “clearness”, enhancing a student”s perception of their ability to remain calm before a formal exam. As such, periodic meditations may make an important psychological impact by assisting to automate one's ability to seek an emotional balance (e.g., seeking to experience a state of tranquility) to place negativities (e.g., angst). For example, a 15-min “walking meditation” practice may help instill a sense of “clearness”, enhancing a student's perception of their ability to remain calm before a formal exam. As such, periodic meditation practices may have a significant psychological impact by automating one's ability to achieve emotional balance (e.g., cultivating a state of tranquility), thereby placating and managing various types of negativities (e.g., reducing angst).As a result, a person might experience not only a greater awareness of their present thoughts and feelings but also a measured ability to regulate these emotional states, fostering a more serene and centered mental state.

**ii. Introspection and the pursuit of life balance**

This reference emphasizes the importance of *self-reflection*, where an individual introspects to examine their thoughts, emotions, and motivations. We argue that, in this context, a state of mindfulness is closely tied to the practice of introspection. Introspection, we contend, is not merely passive thought; rather, it is an active engagement that allows individuals to gain insight into their deepest inner selves. In our recent writing (63), we proposed that the practice and/or process of introspection involves *self-awareness*, where an individual becomes cognizant of their current thoughts and feelings (e.g., “What am I feeling in this moment?”), and *contemplation*, where they reflect thoughtfully on these inner experiences to foster personal growth for the future.
Introspection, in the context of mindfulness, is more than merely a practice or observation. Instead, we argue that introspection is a positive practice that may enable and/or assist a person to understand his or her deep, inner self—for example, “What am I and how do I differ from others?”. One of the co-authors, a practitioner of Buddhist meditation, recently coined a term known as “meditative-reflection”. According to the co-author, engaging in meditative-reflection represents an intimate process: meditation practice serves to facilitate introspective reflection. The uniqueness of meditative reflection, based on the co-author's personal experience, lies in its ability to use meditation practice and subsequent introspective reflection to gain insights into life's true meaning.

**iii. The quest to experience a state of spirituality**

This reference highlights a personal desire to connect with something beyond the materialistic world, often linked to deeper meaning, purpose, and transcendence. In our teaching, we argue that a state of mindfulness is closely connected to, or can give rise to, higher-order trans-mystical experiences. This premise differs somewhat from existing theoretical perspectives on mindfulness (1, 4, 16), which tend to emphasize the importance of humanistic life experiences—for example, self-awareness within one's contextual surroundings and the cultivation of present-moment awareness in daily life. Our focus, instead, views a perceived sense of mindfulness as a “gateway” to facilitating extraordinary life experiences, such as spirituality, which may transcend the realm of the human psyche.
We prefer the term “spiritual context” over spirituality (64–66), as it refers to a broader “system” or comprehensive “framework” that enables individuals to connect with spirituality. Moreover, our viewpoint differs somewhat, positing that spirituality aligns more closely with notions of the transcendent, the sacred, or something “higher” in life. In this analysis, a spiritual context is essential for experiencing something extraordinary that defies logical explanation. According to one of the co-authors, engagement in meditative-reflective practice may provide “insight” into a spiritual context, potentially giving rise to a distinct “spiritual experience”. This could include, for instance, a perceived sense of being transported to another time-space realm or gaining insight into future time contexts. We argue that this premise regarding the relationship between mindfulness and the spiritual context is significant, as it offers an alternative perspective on the nature of mindfulness. In this view, mindfulness is not merely a psychological state but rather a “pathway” to something higher and trans-mystical.

The above discussion illustrates the significance of “mindfulness” experiences, highlighting their profound impact on individual growth. In this context, our proposition suggests that mindfulness is far more complex than its current understanding (1, 19, 41), particularly the notion that it is solely psychological in nature. Our recently published theoretical premise reflects a concerted effort to unify two distinct historical and sociocultural contexts: the Western and the Eastern. The Western context, shaped by its emphasis on individualism, scientific reasoning, and psychological frameworks, typically approaches mindfulness from a psychological perspective, often focusing on educational outcomes, mental health, and wellbeing. Moreover, as we noted earlier, the Western perspective and context have also incorporated relevant methodological approaches to precisely measure and assess mindfulness and the effectiveness of meditation practices. In contrast, the Eastern context, deeply rooted in philosophies such as Buddhism (7, 8), Confucianism (9, 10), and Taoism (11), views mindfulness as part of a broader spiritual and meditative practice, emphasizing the transcendent, the sacred, and trans-mystical experiences.

It is worth noting that numerous researchers align with the Eastern context in their exploration of mindfulness. Both theoretical and empirical studies within this framework seek to deepen our understanding of mindfulness by referencing and drawing upon the rich teachings of Buddhism (57, 62, 67). These researchers often emphasize the spiritual and meditative aspects of mindfulness (e.g., meditation practice may enable a perceived connection with the transcendent), exploring its roots in Buddhist philosophy and practices (e.g., Buddhist meditation). Such studies aim to uncover the transformative potential of mindfulness not just as a psychological tool (e.g., using a perceived state of mindfulness to help alleviate angst), but as a spiritual discipline that fosters self-awareness, personal growth, and a deeper connection to the transcendent and the sacred.

### 3.1 Teaching practices

Our teaching and research activities with both undergraduate and postgraduate students have extensively explored various dimensions of mindfulness. These efforts focus on: (i) utilizing robust methodological designs to accurately measure and assess mindfulness, ensuring clarity and reliability in its evaluation (e.g., using Likert-scale measures with causal modeling techniques); (ii) applying advanced statistical techniques, such as exploratory factor analysis (EFA) and confirmatory factor analysis (CFA), to validate and refine the conceptual understanding of mindfulness as a multifaceted construct; (iii) examining diverse theoretical frameworks, contrasting perspectives from Western epistemology, which often emphasizes psychological and scientific paradigms, with Eastern epistemology, rooted in spiritual and philosophical traditions; and (iv) investigating the relationship between mindfulness and the concept of human agency (68, 69), emphasizing how meditation practices (e.g., Buddhist meditation) can empower individuals to navigate through life with intentionality, autonomy, and self-awareness.

One co-author has been deeply immersed in the practice of Buddhist meditation, drawing from this intimate experience not only to enrich his teachings with students but also to extend its benefits to the broader community. His involvement includes contributing to *life* and *death education* (12–14), a field that emphasizes the concept of “knowledge transformation” (15)—the application of life wisdom (e.g., spiritual wisdom) into practical tools for effective living and preparation for life's final stages. In this context, the co-author makes home visits, offering his insights on mindfulness and Buddhist sutras to individuals nearing the end of life. Through these visits, he seeks to provide comfort, spiritual guidance, and a framework for peace and final acceptance of death. By sharing teachings from Buddhist meditation (7, 70), he helps individuals explore profound questions about existence, cultivate inner calm, and embrace the transition with a sense of meaning and tranquility. This practice not only exemplifies the application of mindfulness in real-world contexts but also highlights its transformative potential in addressing universal human experiences, such as life, death, and the pursuit of spiritual wisdom (e.g., self-transcendence).

## 4 Summation

In summary, the study of mindfulness is a multifaceted and nuanced field, shaped by variations in epistemology and philosophy that offer distinct yet complementary perspectives. Western philosophy often approaches mindfulness through a scientific and psychological lens, emphasizing its practical applications in areas such as education, mental health, wellbeing, and personal development. Grounded in individualism and empirical reasoning, Western frameworks prioritize measurable outcomes—such as improved cognitive function and academic performance—often employing structured methodological approaches. In contrast, Eastern philosophy, rooted in traditions like Buddhism, Confucianism, and Taoism, views mindfulness as deeply interwoven with spirituality, self-awareness, and the pursuit of altruistic goals, such as the attainment of self-enlightenment. Eastern perspectives emphasize the transformative potential of meditative practices (e.g., walking meditation), which not only enhance self-reflection but also foster profound connections to the transcendent and the sacred, offering richer, more complex human experiences.

## 5 Humanistic life experiences

Over the years, our teaching and research endeavors have led us to consider a significant inquiry: the development of a comprehensive theoretical model aimed at providing a holistic understanding of human agency (68, 69). One aspect of this inquiry, as discussed in this theoretical-conceptual article, is how a humanistic understanding (71–73) of life may help explain the nature of mindfulness. To the best of our knowledge, we are among the first researchers to explore this conceptualization. We propose that our work represents a novel approach, bridging multiple fields of research—specifically, life and death education and mindfulness. Central to our viewpoint is the idea that humanistic life experiences are a key component of human agency (68, 69). Within the framework of human agency, individuals can make choices, exercise free will, and shape their life path, especially when confronting existential realities such as mortality.

A “humanistic life experience”, situated within the framework of humanistic psychology (71, 72, 74), refers to an individual's exploration and understanding of core human values, such as *self-awareness, introspection*, and the *pursuit of meaningful experiences*. For example, a teenager may reflect and ask themselves questions such as, “What do I truly desire in life?”, “What do I want to achieve?”, and “Does material wealth matter?” Through such self-contemplation, individuals strive to discover and align with their deep, inner “selves” (75), seeking self-fulfillment, personal growth, and a sense of purpose in life. From our perspective, true “humanistic desires” are inherently altruistic, reflecting an individual's fundamental quest to attain various forms of altruistic fulfillment. Such altruistic fulfillment extends beyond personal gain (e.g., accumulating immense financial wealth), focusing instead on fulfilling a deeper sense of purpose, personal growth, and contributing to the wellbeing of others.

We acknowledge that humanistic life experiences can often seem utopian and unattainable. The realities of human existence—such as suffering, tragedy, personal conflicts, economic instability, and social unrest—make it challenging for many to fully pursue or realize these idealized experiences. For example, why would a social worker pursue “self-enlightenment” (31–33) while being inundated with work? As one of our colleagues recently noted, those most capable of embodying such altruistic ideals may be individuals who have renounced worldly attachments, such as Buddhist monks and nuns, Catholic priests and nuns, and others who live a life devoted to spiritual practice and are perhaps freed from everyday struggles. These individuals, removed from many of life's struggles, may inhabit a different domain, one that allows them to transcend the human plights that limit the rest of us. However, despite these obstacles, we also recognize that the successful attainment of humanistic life experiences remains a worthy life ideal for consideration—something to strive for, even if it is not always fully realized.

Our teaching focus and research endeavors in life and death education (12–14) have deepened our interest in exploring the impact of humanistic experiences on life. Central to this exploration is the acknowledgment that such experiences are intricately linked to cultural and philosophical traditions. Two of the co-authors, both Taiwanese scholars residing in Taiwan, have contributed valuable perspectives and insightful narratives that help enrich the team's collective effort. Taiwan, a society deeply influenced by Buddhism (7, 8), Confucianism (9, 10), and Taoism (11), embodies cultural values that emphasize respect for elders, mutual support, and the practice of compassion. These philosophies cultivate life qualities like “life care”, kindness, and generosity, which are embedded in daily interactions and societal expectations. As a result, many Taiwanese encounter opportunities for humanistic reflection and engagement regularly, whether through caring for family members, participating in community welfare, or practicing rituals of respect and gratitude.

We contend that cultural values, customary practices, and specific expectations cultivate an environment where individuals' humanistic experiences (e.g., personal journeys of self-enlightenment) can flourish. For example, as previously described, it is common for many Taiwanese to participate in communal rituals, engage in charitable activities, and offer support during significant life events such as births, weddings, and funerals. These practices not only enrich personal lives but also strengthen the social fabric of Taiwan, helping to foster a sense of unity, identity, and mutual care. By examining these cultural dimensions, our research seeks to illuminate how humanistic experiences, as shaped by philosophical traditions, influence both individual growth and collective wellbeing. Notably, some of our colleagues collaborating on this research are Taiwanese scholars. Their experiences and narratives provide a compelling case study of Taiwan, illustrating how deeply rooted cultural values can inspire a society to embrace compassion, interconnectedness, and purposeful living. These insights enrich our understanding of life and death education and offer a valuable model for integrating humanistic principles into diverse sociocultural contexts.

## 6 Life and death education

We now turn our attention to a related topic, briefly mentioned earlier, known as *life* and *death education* (12–14). Notably, life and death education can also be differentiated into “life education” (76–78) and “death education” (79–81). Life and death education, as a whole, is a field of study we have explored both individually and collaboratively. Beyond our current research inquiry, which explores the relationship between mindfulness and life and death education, we have pursued other related investigations that demonstrate the transformative potency of this subject. For instance, we have examined how life and death education can provide insights into a person's wellbeing (63), highlighting its capacity to encourage deep self-awareness, introspection, and contemplation of oneself. These inquiries underscore the significance of life and death education research on individual growth and holistic understanding of human agency (68, 69).

Guided by our interest, knowledge, and understanding of Buddhism (7, 8), Confucianism (9, 10), and Taoism (11), our approach to teaching and researching life and death education has taken on a distinctly humanistic perspective. Additionally, we have drawn upon *philosophical psychology* (82, 83) to deepen our interpretation of life and death education, employing a method known as *theoretical infusion* (15). This approach integrates philosophical insights with psychological principles to enrich our understanding of life and death education. We have extensively explored and written about life and death education in our previous works [e.g., (12, 15, 63)], addressing its multifaceted dimensions from theoretical, empirical, and philosophical perspectives. Readers interested in a more detailed discussion are encouraged to refer to our earlier publications [e.g., (63) provides a comprehensive overview], which elaborate on the foundational concepts, practical frameworks, and broader implications of life and death education. In this article, we will provide a concise overview of specific theoretical aspects that are directly relevant to the context of our proposition.

### 6.1 Life education

*Life education* (76, 77, 84) is a positive discipline that explores the concept of “quality functioning”. Quality life functioning reflects one's state of motivation, aspiration, and proactive engagement in striving for various life trajectories. A “life trajectory” is defined as a specific course of action or path that an individual chooses to pursue at a particular moment in time. Over the course of a person's lifetime, he or she has multiple life trajectories—for example, balancing the trajectory of being a part-time university student studying History with that of being a full-time bank employee. Life education teaching, in this context, suggests that the successful attainment of a specific life trajectory, such as a university student completing their undergraduate degree, reflects the proactivity inherent in quality life functioning.

Life trajectories for attainment or accomplishment vary widely, influenced by factors such as personal beliefs, societal expectations, cultural values, and individual life circumstances. These differences reflect the unique ways individuals prioritize their goals and aspirations, shaped by their environment, upbringing, and the collective norms of the communities they belong to. In line with the premise of humanistic experiences, we contend that desirable or quality life trajectories may be closely associated with two contrasting pursuits: “altruistic” *vs*. “non-altruistic”. Altruistic endeavors are driven by a sincere concern for the wellbeing of others, embodying a blend of quality life characters (e.g., trustworthiness) and core life values (e.g., gratitude). These endeavors are reflected in activities such as volunteering for community projects, mentoring others, or engaging in acts of kindness that contribute to the collective welfare of society. In contrast, non-altruistic endeavors center on personal growth, self-fulfillment, and the pursuit of individual aspirations. These pursuits often align with goals such as achieving financial wealth, gaining social recognition, or attaining materialistic milestones. That being said, we acknowledge a degree of bias and subjectivity in both our teaching practices and personal beliefs. Our focus tends to lean heavily toward emphasizing altruistic endeavors, often at the expense of exploring and validating the importance of non-altruistic pursuits. This overemphasis reflects our commitment to fostering a culture of compassion and collective responsibility, but it may inadvertently downplay the significance of individual aspirations and self-oriented achievements.

One notable aspect of life education teaching is its distinctive emphasis on personal philosophization, encouraging individuals to explore open-ended questions about the purpose and meaning of life. Questions such as “What is the true purpose of life?”, “What goals are worth striving for?”, and “Can personal resolve help one navigate and overcome life's challenges?” may serve as facilitators for daily introspection, encouraging individuals to reflect deeply on their values, beliefs, aspirations, and inner resilience. This reflective approach highlights the significance of personal philosophy, uniquely shaped by an individual's experiences and perspectives. Unlike disciplines such as physics, which often seek definitive answers, life education embraces open-ended and inconclusive discourse as a fundamental principle. For instance, the question “What is the main purpose of life?” is not meant to have a singular, universally accepted answer but rather to initiate personal exploration, highlighting the subjective nature of such inquiries. Through this process of philosophization, life education teaching aims to encourage individuals to think deeply and expansively about life' certainties and uncertainties.

### 6.2 Death education

*Death education* (79–81) is a discipline that focuses on the inevitable end of life, as well as related topics such as loss, grief, and mourning (85, 86). From personal experience, we acknowledge that, compared to other subject areas (e.g., Cognitive Psychology), death education is often perceived as uncomfortable or unpleasant to teach and study. If given a clear choice, many individuals would likely choose life education over death education, as the latter often confronts difficult and emotionally charged subjects. While some may disagree with this sentiment, the study of palliative care (79, 87), such as Last Aid Courses (88, 89) designed to assist those facing end-of-life decisions, as well as the exploration of loss and grief, can be profoundly challenging and emotionally taxing. These topics often involve navigating difficult emotions and confronting harsh realities, which can make them less appealing to engage with, even though they are critically important.

Over the years, we have made concerted efforts to innovate the teaching of death education. Rather than focusing solely on its pragmatic aspects—such as the provision of end-of-life care for individuals nearing death—we have recently developed a theoretical framework to bring a more scholarly, systematic approach to the subject. This framework, which we term the “CPPPS Framework of Death Education”, seeks to deepen our understanding of death by incorporating five distinct theoretical lenses. These lenses provide a multifaceted view of death education, allowing for a more holistic exploration of the topic. In brief, the five lenses are as follows:

i The cultural lens (c):
  This lens highlights the significance of historical and sociocultural diversity in shaping perspectives and interpretations of death and dying across different cultures and societies. For instance, practices such as ancestor worship (90, 91), observed in some cultural groups (e.g., Chinese), exemplify unique rituals that reflect these diverse viewpoints.

ii The philosophical lens (p):
  This lens delves into personal philosophies of death and dying, fostering a deeper engagement with introspection, self-awareness, and contemplation about the meaning and nature of death and death-related matters. For instance, some individuals and cultural groups may reflect on the possibility of life after death, considering concepts such as the existence of an afterlife (92, 93) or reincarnation (94, 95). This process invites a nuanced understanding of how people relate to death on a deeply personal level, shaped by their unique cultural, spiritual, or philosophical frameworks.

iii The pragmatic lens (p):
  This lens emphasizes the practical dimensions of death education, highlighting the critical nexus between theoretical knowledge and applied practice. It seeks to connect theoretical understanding with real-world applications, ensuring that individuals and professionals are equipped to navigate the complexities of death and dying in meaningful ways. For instance, a prominent example of practicality in palliative or end-of-life care (89, 96, 97) is the implementation of quality educational initiatives, such as Last Aid courses (88, 89). These courses provide essential training on topics like compassionate communication, pain management, and psychological support for individuals nearing the end of life and their families.

iv The psychological lens (p):
  This lens explores the development of a positive “psychological persona”, which may assist individuals in learning how to cope with the emotional and psychological challenges associated with death, loss, and grief. It emphasizes the importance of fostering an appropriate mindset that can help individuals navigate difficult times. By the same token, it is important for one to be able to cognitively process grief and know how to regulate negative emotions during times of loss. A positive psychological persona—such as maintaining an optimistic outlook on life or possessing a strong sense of personal resolve—can help individuals effectively manage negative emotions like grief, angst, or despair.

v The sociological lens (s):
  This lens examines death and dying from a societal perspective, exploring how different societies perceive, handle, and discuss death, and how death rituals and practices influence collective understanding. Unlike other theoretical lenses, such as the philosophical lens, the sociological lens focuses on the relationship between the individual and society, which we term as the “individual-society” dynamic. This lens highlights how societal norms, values, and expectations shape personal experiences of death and influence behavior during times of loss. For example, societal expectations may compel individuals and their families to adhere to certain rituals, customs, or behaviors during mourning, funerals, or the grieving process, reflecting the way society influences personal responses to death.

Our approach to teaching death education incorporates all five lenses, creating a comprehensive and multifaceted framework for understanding death. The use of these lenses is complementary, enriching the learning experience by offering diverse perspectives. For instance, when students engage with video resources depicting real-life cases of patients in need of palliative care, they experience the pragmatic lens in action, which emphasizes the practical aspects of death and end-of-life care. In contrast, group discussions during tutorial sessions, where students explore open-ended questions such as “*Where does one's state of consciousness go after death?”*, highlight the philosophical lens, prompting deep reflection on the nature of mortality and the mysteries surrounding it. Similarly, introducing students to various rituals and customs, such as the practice of ancestor worship, illustrates the significance of the cultural lens, highlighting how death-related beliefs and practices shape collective understanding across different societies. This teaching involving multiple lenses ensures that students not only gain practical knowledge but also engage with the emotional, psychological, and existential dimensions of death, fostering a holistic understanding of this complex topic.

### 6.3 Life education + death education: in totality

Life education (76, 77, 84) and death education (79–81) are distinct disciplines typically taught as separate subjects in academic settings (e.g., courses like “Life Education: An Introduction”). Similarly, research often treats them as independent fields of study. For instance, Shu, Miao (80) examined the growing need for death education in China, while Seng and Lee (81) explored the challenges associated with implementing death education in Malaysia. Despite this separation, our work frequently acknowledges and explores the interconnectedness between life education and death education, emphasizing their intimate relationship and the ways they complement and inform each other in both theoretical and practical contexts. We propose that to truly understand death, one must first grasp the principles of life education. Similarly, the teaching of death education can foster a deeper appreciation for life, highlighting the interconnectedness of the two disciplines.

## 7 Life, death, and mindfulness

We now turn our attention to an important area of inquiry: *the relationship between life and death education and mindfulness*. Through years of teaching and research, we have come to consider this relationship as potentially reciprocal. On one hand, teaching life and death education (14, 76, 84) may deepen our understanding of mindfulness by shedding light on its significance in navigating existential questions. On the other hand, the multifaceted nature of mindfulness—particularly its philosophical and spiritual dimensions (58)—can enrich our appreciation of the humanistic aspects of life and death, offering profound insights into their interconnectedness. At the core of our thesis lies an emphasis on the significance of humanistic life experiences. Such life experiences encompass a wide range of *meaningful and transformative endeavors*, including:

i The self-fulfillment of altruistic actions:

Engaging in acts of kindness and generosity not only benefits others in society but also serves as an important source of self-fulfillment and personal growth. When individuals help others, whether through small gestures or significant efforts (e.g., a teenager volunteering at a homeless shelter), they often experience a sense of purpose and personal gratification. This self-fulfillment arises from the knowledge that their actions have positively impacted someone else's life, fostering a sense of connection and life meaning. As individuals engage in acts of altruism, they contribute to both the wellbeing of others and their own life trajectory toward self-actualization.

ii Exploration of self-awareness and introspection:

Exploring the deeper dimensions of one's inner self fosters an in-depth understanding of oneself, encouraging the engagement of self-awareness and introspection. This process promotes a thoughtful and contemplative approach to life, allowing individuals to reflect on their values, beliefs, and emotions. By delving into their inner selves, individuals can identify their latent strengths, address personal challenges, and gain clarity about their aspirations and purpose in life. This life trajectory of self-discovery and personal understanding not only facilitates personal growth, but also enhances the ability to navigate life's complexities with greater resolve and mindfulness.

iii Striving for human perfection and utopian ideals:

Aspiring toward life ideals involves striving to create a harmonious and peaceful society for all. This pursuit entails not only addressing immediate social issues but also promoting long-term values of equality, compassion, and mutual respect. It seeks to build a world where individuals can thrive in a supportive environment, free from inequality, discrimination, and human suffering. These altruistic aspirations inspire efforts to improve social, economic, and environmental conditions, ultimately contributing to the overall advancement of human wellbeing and existence.

iv Expressing and imparting virtues:

Possessing life characteristics (e.g., trustworthiness) and life values (e.g., gratitude) is an important development that helps a person understand life's true meaning. Moreover, positive life characteristics and values provide a foundation for individuals to make thoughtful, ethical decisions and build meaningful relationships with others. When individuals embrace these traits, they gain a deeper understanding that enables them to navigate life's challenges with personal resolve and determination. Ultimately, these life qualities (e.g., gratitude) guide people toward living a more fulfilling and interconnected life, grounded in altruistic and moral clarity.

In summary, the above endeavors inherently humanistic and personal, underscoring the potential connection between mindfulness (1, 58, 67, 70) and life and death education teaching (12–14). Our proposition, in this analysis, argues that there is a significant degree of overlap in the knowledge and understanding of the two subject disciplines, akin to a Venn diagram. This overlap suggests that the insights and frameworks of mindfulness and life and death education are not entirely distinct but rather interrelated, with shared concepts that enrich the study and practice of both fields. We contend that there are two possible explanatory accounts that support this conceptualization.

First, as we outlined earlier, the comprehensive study of mindfulness—encompassing its philosophical, psychological, and spiritual dimensions—provides valuable insights into a person's holistic life experiences, including deeply spiritual and, at times, philosophical aspects. This understanding enables individuals to recognize the higher-order, altruistic essence of life's functioning. In this context, daily functioning transcends the basic or straightforward elements of life (e.g., one's motivation to study or seek social recognition). Mindfulness, therefore, encourages individuals to pursue more profound experiences, such as a deep desire to connect with the environment. Meditation practice, in this regard, serves as a gateway to encountering such humanistic experiences. Furthermore, we argue that meditation and a cultivated sense of mindfulness provide a solid foundation for individuals to approach death, loss, and grief from a more positive perspective.

Second, life and death education teaching provides various theoretical perspectives (e.g., the philosophical lens) that complement each other, offering enriching insights for a deeper understanding of life's functioning. A key feature of life and death education is its focus on humanistic life experiences, encouraging individuals to engage in philosophical reflection and consider the importance of balancing altruistic and non-altruistic life ideals. We argue that this approach advocates for prioritizing self-actualization, personal growth, and meaningful relationships over the pursuit of material wealth. By fostering an understanding of life's deeper purpose, it invites individuals to reflect on the positive value of humanistic experiences, such as pursuing altruistic goals (e.g., choosing self-actualization). We assert that this teaching engages the philosophical, psychological, and spiritual dimensions of mindfulness.

We acknowledge, however, that our theoretical premise (e.g., the endeavor to strive for human perfection and utopian ideals) remains in its formative stages, representing an initial effort to conceptualize a novel integration of ideas. The present article in this sense aims to advance a seminal proposition, grounded in careful philosophical analysis, that establishes the groundwork for future empirical investigation. Rather than offering conclusive claims or presenting evaluative data, this work is intended to stimulate critical reflection and invite scholarly dialogue from readers. In this sense, we view the article not as a definitive account but as a generative contribution—an opening gesture toward a broader conversation that we hope will inspire further theoretical refinement and practical inquiry.

## 8 Illustrative curriculum concepts based on the life + death and mindfulness framework

We acknowledge that while our proposed conceptual synthesis, as overviewed and discussed, is still under development, we outline a *hypothetical curriculum* (63, 98) that may reflect the integration of mindfulness with life and death education. This integration is envisioned not merely as an academic exercise and/or example but as a potential contributor to holistic wellbeing. By fostering deeper awareness of mortality and meaning, the proposed curriculum seeks to enhance dimensions of optimal health functioning—including emotional regulation, existential coherence, and compassionate engagement with self and others. These illustrative concepts, we contend, may serve as a blueprint for future implementation and evaluation within educational or therapeutic contexts that aim to promote flourishing in the face of life's impermanence. We see this curricular design as offering a pedagogical framework for nurturing psychological resilience, cultivating prosocial behavior, and supporting the development of a coherent life narrative—all of which are widely recognized as facilitators of optimal health.

*Course Title*: Mindfulness, Mortality, and Meaning:

*Learning Objectives*:

- Cultivate an experiential and philosophical understanding of mindfulness as a foundation for self-awareness and mental wellbeing.
- Engage in personal introspection and dialogue on life trajectories and mortality to foster existential clarity and emotional depth.
- Examine the moral, cultural, and spiritual dimensions of altruism, impermanence, and compassion as components of an integrated approach to human flourishing.

*Sample Modules*:

- Meditation as Self-Inquiry: Daily guided meditation practices, paired with journaling exercises reflecting on emotional and cognitive states, aimed at enhancing self-regulation and introspective capacity.
- Dialogues on Death: Small group discussions analyzing cultural rituals and personal beliefs surrounding death, promoting empathy, cultural literacy, and existential awareness.
- Life Purpose Narratives: Students draft personal statements exploring life goals and meaning, informed by meditative-reflective practices, to support identity coherence and future-oriented thinking.
- Mindfulness and Service: Exploration of altruism through community service activities, followed by reflective essays examining the interplay between mindful presence and ethical action.

*Assessment Tasks*:

- Reflective essays analyzing students' meditation experiences and emotional insights, with attention to themes of growth, struggle, and awareness.
- Case study analysis connecting life-and-death themes with mindfulness theory, encouraging the application of abstract concepts to lived realities.
- Final project: a personal life manifesto that articulates one's evolving understanding of mindful living and mortality, framed as a dynamic guide for meaningful and health-promoting engagement with life.

Overall, then, our sample example above illustrates a formative yet conceptually rich approach to integrating mindfulness [e.g., (1, 24, 62)] with life and death education [e.g., (12–14)] in support of optimal health functioning. Grounded in philosophical inquiry and experiential learning, the sample curriculum encourages emotional insight, existential reflection, and compassionate action—elements widely associated with psychological resilience and human flourishing. Through modules on meditation, mortality, purpose, and service, it cultivates mindful self-awareness alongside a deeper engagement with life's impermanence. While still hypothetical, we contend that this framework offers a promising foundation for future empirical research and practical implementation, positioning mindfulness not only as a personal practice but as a transformative educational pathway toward holistic wellbeing.

## 9 Toward transferability and implementation

Recognizing the conceptual nature and broader significance of our work, we propose several guiding principles to support future adoption, adaptation, and evaluation of the theoretical premise across different contexts. While grounded in a humanistic (71–73) and philosophical (15, 75, 82) approach, this initiative has critical implications for public health. By promoting mental resilience, reflective self-awareness, and meaning-oriented discourse, for example, it aligns with global efforts to enhance personal wellbeing and mitigate psychological distress—particularly in response to rising rates of suicide (99), burnout (100), and existential despair across diverse populations (101). By the same token, of course, it is an internal desire for one to aspire toward and live a meaningful and self-fulfilling life. As such, we contend that our theoretical premise—pertaining to the relationship between mindfulness and life and death education—may contribute meaningfully to emerging strategies for population-level mental health promotion through formal education. A growing body of literature underscores the value of embedding preventive and meaning-oriented frameworks within educational settings to foster emotional resilience, reflective awareness, and psychosocial wellbeing (102, 103). Global initiatives, such as those led by the World Health Organization (104) and The Lancet Commission (105), advocate for integrative, cross-sectoral approaches that align mental health promotion with educational, cultural, and developmental goals. These strategies are especially critical in light of rising psychological distress across diverse populations and the urgent need for scalable, context-sensitive interventions (106). As an example, then, consider the following sample:

*Instructor Guidelines–*

*i Facilitative Pedagogy for Mental Safety*:

Instructors are encouraged to adopt a facilitative rather than didactic stance, prioritizing the creation of psychologically safe, inclusive, and trauma-informed learning environments. These spaces enable students to engage in reflective and emotionally sensitive discourse—a process increasingly recognized as essential for preventing emotional isolation and enhancing protective factors for mental health.

ii *Mindfulness Practices Aligned with Philosophical Themes*:

Weekly structured meditation practices and journaling prompts should be integrated into the curriculum and thematically aligned with philosophical inquiries into impermanence, meaning, and compassion. These core themes are empirically linked to improved emotional regulation, reduced stress, and enhanced capacity for existential acceptance—all of which are central to current public health models of mental wellbeing and suicide prevention.

iii *Philosophical Inquiry and Existential Dialogue*:

The use of open-ended questioning techniques invites students into deeper philosophical engagement and existential reflection. Such approaches cultivate cognitive flexibility, self-awareness, and existential insight, which emerging evidence identifies as protective against despair, rigid thinking patterns, and suicide ideation. Facilitating these forms of discourse may play a preventive role by nurturing students' capacity to meaningfully confront uncertainty, loss, and life transitions.

*Pilot Programs*–

i *Embedding in Professional and General Education Contexts*:

This framework can be piloted within general education or professional training programs—particularly in fields such as teacher education, counseling, social work, or health sciences—where there is a pressing need to equip individuals with tools for emotional resilience, empathy, and meaning-centered practice. By fostering intrapersonal and interpersonal understanding among future professionals, such curricula may yield downstream benefits in community mental health and contribute to public health infrastructure focused on early intervention.

ii *Research-Informed Implementation*:

Utilizing case study or action research methodologies allows educators to assess student engagement, emotional development, and shifts in meaning-making capacities. These data can inform continuous refinement of both content and pedagogy, while also building an evidence base for the integration of contemplative and philosophical approaches into public health education strategies.

*Cultural Adaptation*–

i *Locally Responsive Ethical and Spiritual Integration*:

To enhance both relevance and effectiveness, the curriculum should be adapted to reflect the spiritual, ethical, and existential concerns of local communities. This includes incorporating culturally specific teachings that align with learners' lived experiences and moral frameworks—thereby fostering a sense of recognition and belonging that is critical to culturally competent mental health promotion.

ii *Interfaith and Indigenous Worldviews*:

Building upon its current emphasis on Buddhist (7, 107), Confucianist (108, 109), and Taoist (11, 110) perspectives, the framework can be expanded to engage with interfaith, indigenous, and other community-based wisdom traditions. Doing so enhances cross-cultural inclusivity, strengthens relational and spiritual dimensions of wellbeing, and supports population-level mental health strategies that respect diverse cosmologies and coping resources.

Overall, then, we acknowledge that our theoretical tenets so far (e.g., the self-fulfillment of altruistic actions) are philosophical and speculative, requiring further empirical substantiation. Nonetheless, our proposed sample is intended not only to demonstrate the pedagogical feasibility of integrating mindfulness with life-and-death education but also to exemplify the broader “universality” and “generalizability” (63, 98) of the present theoretical premise. By offering a flexible, adaptable structure grounded in shared human concerns—such as improved health wellbeing, mortality, compassion, meaning, and resilience—this framework aspires to resonate across diverse life experiences, educational settings, and sociocultural contexts. Whether in secular classrooms (111, 112), interfaith programs (113, 114), or culturally specific training modules (115, 116), the underlying principles can be recalibrated and adapted to align with local epistemologies and community needs. In doing so, our proposition holds relevance and promise as a cross-culturally approach to health promotion and existential education, advancing a vision of wellbeing that is inclusive, reflective, and globally relevant.

## 10 Overview: why study life, death, and mindfulness?

We pose a reflective question for readers to reflect and contemplate: Why do we choose to study life, death, and mindfulness together? Why not approach them as separate inquiries, such as one exploring the effectiveness of mindfulness on its own? To summarize, our teaching and research in life and death education have expanded into related domains, examining how this field can deepen our understanding of personal wellbeing, foster spiritual growth, and promote peacebuilding. For instance, can life and death education be used to foster peace, harmony, and unity? Consequently, we expand our focus to explore a related inquiry: to what extent can teaching life and death education illuminate the fundamental nature of mindfulness? Alternatively, we examine how these two domains—life and death education and mindfulness—might intersect, mutually informing and reinforcing one another in meaningful ways.

Mindfulness (1, 16, 18, 67) and life and death education (12–14), though distinct in focus, share a common purpose as disciplines regarded as “positive” or proactive, aiming to provide an in-depth understanding of life experiences. Together, they provide a theoretical lens that enables individuals to cultivate deeper connections with themselves and others in society. In this context, we view mindfulness and life and death education as humanistic disciplines and sources of insight, capable of fostering hope, inspiring personal growth, and driving societal transformation. By encouraging self-awareness, introspection, spirituality, and compassion, these disciplines contribute to the betterment of individuals and the cultivation of a more harmonious society.

The world is currently facing a series of human crises, including the recent COVID-19 pandemic, ongoing wars in Gaza and Ukraine, the looming threat of a potential China-Taiwan military conflict, and widespread economic and social uncertainties across many regions. Admittedly, our perspective may be somewhat subjective and biased due to our teaching and research interests. Nevertheless, we firmly believe that both mindfulness (1, 16, 18, 67) and life and death education (12–14) are significant and impactful disciplines, offering valuable insights and tools for study and learning in these challenging times. A central thesis of our position is the belief that reflecting on and appreciating the essence of positive humanistic life experiences holds profound meaning. What is the main purpose of life, one may ask? This question is not meant to promote any specific belief or viewpoint but to underscore the potential of mindfulness and life and death education as essential tools for exploration and deeper understanding of life. Engaging in meditation practice, for instance, allows individuals to recognize the aesthetic and meaningful aspects of life. Such practices may lead to self-actualization experiences, fostering a deeper understanding of one's purpose, values, and connection to the world. Similarly, exploring life education emphasizes the importance of pursuing altruistic goals, encouraging individuals to prioritize acts of kindness, compassion, and community-building over purely self-serving objectives.

## 11 Development for consideration

Situating the study of mindfulness within the context of life and death education offers a novel and innovative approach to advancing our understanding of its nature. This perspective not only deepens our insights into mindfulness but also highlights the broader significance of life and death education teaching itself. The present theoretical-conceptual article provides a comprehensive overview of the potential relationship between these two disciplines. By integrating mindfulness with life and death education, this approach fosters a holistic understanding of human nature, highlighting the significance of introspection and the pursuit of meaningful life paths. At the core of our proposition is an emphasis on encouraging individuals to actively seek positive life functioning, cultivating personal growth, wellbeing, and a deeper sense of purpose (12, 15).

### 11.1 Educational implications for development

We contend that our theoretical premise and proposition—exploring the potential relationship between mindfulness and life-and-death education through philosophical analysis—hold daily relevance and offer practical considerations. In this section, we outline several educational implications for consideration, including the importance of *curriculum development, educational training, development of policy*, the use of *workplace and school-based interventions*, and the *practice of community engagement*.

#### 11.1.1 Curriculum development

Educational institutions may wish to integrate elements of life and death education (e.g., a particular theme) (63, 98) into existing mindfulness-based programs (e.g., the Mindfulness-Based Stress Reduction). Doing so may enable a more holistic teaching delivery that incorporates not only cognitive-emotional benefits (e.g., the alleviation of angst) but also deeper philosophical and spiritual reflections on human existence. At the same time, we contend that the integration of mindfulness and life-and-death education—such as merging key elements of mindfulness teaching with core aspects of life education—holds the potential to assist educators in conceptualizing new topical themes, learning outcomes (LOs), and subject content for teaching and learning. This integrative approach encourages the development of innovative pedagogical pathways that are responsive to both cognitive and affective domains of student development. For instance, beyond the established “CPPPS Framework of Death Education”, we have recently proposed a complementary model—the “Life + Death Education Framework” (98)—which aims to holistically address themes of existential awareness, personal meaning, and mindful engagement with life and mortality. Educators can draw upon these frameworks as foundational structures for curriculum design, using them to inform the selection of teaching strategies, the formulation of learning objectives, and the integration of cross-disciplinary content.

#### 11.1.2 Educational training

Teacher education and/or workplace programs may include professional development modules that incorporate meditation and mindfulness practices grounded in both Western (e.g., humanistic) and Eastern traditions, including Buddhism (7, 107), Confucianism (108, 109), and Taoism (11, 110). Preservice and inservice programs (e.g., a semester-long course) may be specifically tailored to cater to individual needs, helping educators, employers, and employees cultivate self-awareness, emotional regulation, and pedagogical sensitivity. Such programs may also encourage reflective practice and support the integration of mindfulness into personal and professional domains, thereby fostering holistic development and wellbeing (e.g., the “Contemplative Pedagogy and Life Education Initiative”, a pilot program designed to blend mindfulness practices with existential and life-and-death themes in teacher training).

#### 11.1.3 Policy formulation

Policymakers, educators, and stakeholders may consider formulating education policies that formally recognize the value of integrating life and death education alongside mindfulness. Notably, in Taiwan, the formal teaching and curriculum development of life-and-death education are officially recognized and mandated within the school system (117, 118). Policies that support the inclusion of these frameworks in K−12 and higher education can promote themes related to life and death education and/or mindfulness, such as character development, emotional resilience, compassionate citizenship, social harmony, and life enlightenment. As an interesting note, one of the universities in Taiwan with which we collaborate has a mandated course known as *Life Enlightenment* that all students, regardless of their degree programs, are required to undertake. This Life Enlightenment course, in brief, reflects the university's commitment to promoting spiritual growth, self-understanding, and an appreciation of the meaning and value of life. It serves as a core component of the institution's broader educational policy, aiming to foster students' holistic development by encouraging reflection on existential themes, emotional awareness, and ethical living.

#### 11.1.4 Workplace and school-based interventions

Schools, organizations, and workplaces can implement mindfulness- and life-related intervention programs that nurture spiritual growth, foster life appreciation, and support character development (e.g., cultivating gratitude), social harmony, and altruistic values. Such initiatives reflect the deeply interconnected nature of life education and humanistic mindfulness, emphasizing not only cognitive and emotional wellbeing but also the moral and existential dimensions of human development. By embedding these principles into everyday practice, these settings can create more compassionate, reflective, and ethically grounded communities. At the same time, we are mindful that this recommendation may not always be feasible in practice. Factors such as time constraints, logistical challenges, limited resources, institutional priorities, and varying perceptions of the usefulness or relevance of such programs can pose significant barriers to implementation. These practical considerations, however, highlight the need for flexible, context-sensitive approaches that allow for gradual integration, adaptation to local needs, and sustained support from leadership and stakeholders.

#### 11.1.5 Community engagement

Drawing from our findings, educational leaders may extend mindfulness and life and death education practices to the broader community. How can the local community benefit from the teaching and learning of mindfulness and/or life-and-death education? We contend that this sample question underscores the importance of everyday relevance and practical application—not only for the individual, but also for the broader community. An interesting observation noted by several of us in Taiwan is the proactive engagement in social and benevolent activities by many Taiwanese individuals—for example, some choose to volunteer at their local Buddhist temple every Saturday. Likewise, as mentioned earlier, one of the co-authors actively collaborates with a non-profit organization, conducting home visits and sharing mindfulness practices and spiritual teachings with individuals nearing the end of life. This recommendation regarding community engagement is a noteworthy endeavor worth considering.

The educational applications outlined here highlight the importance of mindfulness and life and death education, not only as academic disciplines but also as practical tools for real-world transformation. Their integration into school culture and community development initiatives may offer opportunities for enhancing individual wellbeing, fostering social cohesion, and cultivating a more compassionate, mindful society. Central to this thesis, of course, is an awareness of the importance of the public health system as both a structural and cultural force in shaping educational priorities. When mindfulness and life-and-death education are aligned with public health initiatives (e.g., Every Mind Matters^1^, they can contribute to broader goals such as mental health promotion (119), suicide prevention (120), palliative care awareness (121), and emotional resilience building (122). In this way, educational institutions—working in partnership with public health agencies—can play a pivotal role in fostering holistic wellbeing across the lifespan, beginning from early childhood and extending into adulthood. Such collaboration not only enhances the relevance of educational interventions (e.g., the use of Vipassanā meditation) but also reinforces the interconnectedness of health, education, and community flourishing.

### 11.2 Limitations and future directions

While our theoretical-conceptual article offers a robust framework for development, we also acknowledge several limitations that are important to address. Like any research inquiry, it is crucial to recognize that our propositions are not without their constraints. These limitations, for example, include the need for more empirical evidence to support the integration of mindfulness and life-and-death education, as well as the challenges in adapting these ideas across diverse cultural and educational contexts. Furthermore, practical implementation may face obstacles such as time constraints, resource limitations, and varying levels of acceptance or understanding within different educational systems and communities. Acknowledging these limitations is essential for refining the proposed framework and for guiding future research and application:

- Theoretical focus:
  Our article is conceptual in nature and does not include any empirical data, instead relying on the discourse of philosophical analysis. Philosophical inquiries, we contend, are open-ended, with subjective interpretations and non-definitive answers. For example, our recently developed multifaceted model of mindfulness (37, 58) is largely philosophical and speculative in nature, with certain elements or aspects that are somewhat difficult to empirically validate. In a similar vein, the CPPPS Framework of Death Education, though seminal in its evolution, remains theoretical and calls for further refinement, including empirical development and validation. As a result, future studies may seek to validate our propositions through qualitative or mixed-method approaches (123, 124), such as case studies or narrative analyses. Researchers may wish to consider exploring lived experiences of individuals who engage deeply with meditation practices (e.g., “Walking meditation”) in contexts that explicitly address life and death themes—such as palliative care settings, spiritual retreats, or philosophical life education programs. These qualitative accounts, using the methodology of “reflective-meditative documentation” (15, 125) could offer insight into how mindfulness fosters existential awareness, acceptance of mortality, and a reconfiguration of meaning-making. In a similar vein, empirical studies could examine how engagement with death education—such as appreciating the significance of rituals like ancestor worship (90, 91)—may influence the cultivation or deepening of mindfulness, particularly in relation to presence, the impermanence of life, and the practice of non-attachment. Such a bi-directional inquiry invites attention to the mutually reinforcing relationship between reflective awareness and existential pedagogy. While theoretical propositions such as ours lay the groundwork, empirical exploration—through interviews, ethnographies, or longitudinal case studies—could shed light on how these abstract constructs take shape in real-world contexts. Ultimately, we argue that the relationship between mindfulness and life and death education is dynamic and context-sensitive, shaped by cultural, spiritual, and personal dimensions that resist simplistic categorization. As such, the work of philosophical modeling and empirical validation must proceed in tandem, informing and evolving alongside one another.
  Alternatively, researchers may choose to refine our existing premises—such as the multifaceted model of mindfulness (37, 58)—and explore comparable conceptualizations or alternative models that either resonate with or counter our propositions. This may include potential expansions or reinterpretations of the Life + Death Education Framework (63, 98), particularly in relation to cultural, spiritual, or developmental contexts. We contend that such scholarly engagement not only reinforces the value of philosophical psychology but also contributes to the evolving conceptual understanding of how mindfulness and life and death education are defined, contextualized, and operationalized across diverse epistemological and pedagogical traditions.

- Cultural specificity:
  Much of our argumentation thus far [e.g., (12, 75, 98)] is grounded in Eastern philosophical traditions, particularly those rooted in Taiwanese cultural contexts and the teachings of Buddhism (7, 107), Confucianism (108, 109), and Taoism (11, 110). While this focus offers rich insights for understanding—such as the importance of cultural diversity, spiritual cultivation, and holistic wellbeing—it does, however, present certain limitations. Chief among these is the potential lack of generalizability to non-Eastern or secular contexts, where differing cultural, philosophical, or religious orientations may shape perceptions of mindfulness and life and death education in distinct ways. For example, our argument and theoretical premise regarding the trans-mystical nature of mindfulness—namely, that mindfulness is not merely a psychological state but a “pathway” to something higher and transcendent—may not be readily received or fully appreciated within Western contexts. As such, then, we contend that future theoretical and empirical explorations may consider broader, cross-cultural comparisons to examine the applicability and adaptability of these frameworks across diverse settings.

- Lack of practitioner perspectives:
  Our analysis primarily reflects academic and philosophical perspectives. As scholars, we are intentional in our approach, particularly in the development of new theoretical models for dissemination. We draw on existing research, including our own, as well as our interpretations, insights, and philosophical reflections, with the aim of exploring new frontiers in research. As such, we contend that there may well be a misalignment or dissonance between theory and practice. For example, our multifaceted model of mindfulness was developed using a Delphi methodology (126, 127), involving colleagues with strong scholarly expertise in the areas of meditation and mindfulness. What we propose for teaching, then, may not necessarily translate into daily relevance or practical application. Incorporating the narratives of educators, students, and administrators—through interviews or focus groups—could offer a more grounded understanding of how the relationship between mindfulness and life and death education functions in real-world contexts.

- Potential bias:
  As acknowledged throughout, our commitment to humanistic and altruistic values may influence the interpretation and emphasis of certain arguments. Moreover, as noted earlier, the use of philosophical analysis as a mode of inquiry presents certain limitations—philosophical inquiries are inherently open-ended and often give rise to ontological subjectivity (128, 129). Our collective interest in Eastern philosophical traditions, as described, has led us to place a strong emphasis on understanding spirituality in a trans-mystical or metaphysical sense (125). Such an approach may, in turn, shape our reasoning and influence the conclusions we draw, potentially limiting the scope of our analysis to perspectives rooted in these traditions. This focus, while offering valuable insights, might not fully account for alternative philosophical or cultural viewpoints that challenge or reinterpret the metaphysical aspects of mindfulness and life-and-death education. Consequently, we acknowledge the need for broader, more inclusive perspectives that consider other global and secular frameworks for future development.

Overall, then, we invite future researchers to empirically investigate the interrelationship between mindfulness and life and death education, assess the impact of their integration on personal and educational outcomes, and refine the proposed premise to accommodate a broader range of perspectives and applications.

## 12 Conclusion

Situating the study of mindfulness within the context of life and death education offers a novel and innovative approach to advancing our understanding of its nature. This perspective not only deepens our insights into mindfulness but also highlights the broader significance of life and death education teaching itself. The present theoretical-conceptual article provides a comprehensive overview of the potential relationship between these two disciplines. By integrating mindfulness with life and death education, this approach fosters a holistic understanding of human nature, highlighting the significance of introspection and the pursuit of meaningful life paths. At the core of our proposition is an emphasis on encouraging individuals to actively seek positive life functioning, cultivating personal growth, wellbeing, and a deeper sense of purpose.

## Data Availability

The original contributions presented in the study are included in the article/supplementary material, further inquiries can be directed to the corresponding author.

## Footnotes

^1^Source: https://www.nhs.uk/every-mind-matters/)
